# Characterization of Multiwalled Carbon Nanotube-Reinforced Hydroxyapatite Composites Consolidated by Spark Plasma Sintering

**DOI:** 10.1155/2014/768254

**Published:** 2014-03-04

**Authors:** Duk-Yeon Kim, Young-Hwan Han, Jun Hee Lee, Inn-Kyu Kang, Byung-Koog Jang, Sukyoung Kim

**Affiliations:** ^1^School of Materials Science and Engineering, Yeungnam University, Gyeongbuk 712-749, Republic of Korea; ^2^Department of Advanced Materials Engineering, Dong-A University, Busan 604-714, Republic of Korea; ^3^Department of Polymer Science and Engineering, Kyungpook National University, Daegu 702-701, Republic of Korea; ^4^Advanced Ceramics Group, National Institute for Materials Science, Tsukuba, Ibaraki 305-0047, Japan

## Abstract

Pure HA and 1, 3, 5, and 10 vol% multiwalled carbon nanotube- (MWNT-) reinforced hydroxyapatite (HA) were consolidated using a spark plasma sintering (SPS) technique. The relative density of pure HA increased with increasing sintering temperature, but that of the MWNT/HA composite reached almost full density at 900°C, and then decreased with further increases in sintering temperature. The relative density of the MWNT/HA composites increased with increasing MWNT content due to the excellent thermal conductivity of MWNTs. The grain size of MWNT/HA composites decreased with increasing MWNT content and increased with increasing sintering temperature. Pull-out toughening of the MWNTs of the MWNT/HA composites was observed in the fractured surface, which can be used to predict the improvement of the mechanical properties. On the other hand, the existence of undispersed or agglomerate MWNTs in the MWNT/HA composites accompanied large pores. The formation of large pores increased with increasing sintering temperature and MWNT content. The addition of MWNT in HA increased the hardness and fracture toughness by approximately 3~4 times, despite the presence of large pores produced by un-dispersed MWNTs. This provides strong evidence as to why the MWNTs are good candidates as reinforcements for strengthening the ceramic matrix. The MWNT/HA composites did not decompose during SPS sintering. The MWNT-reinforced HA composites were non-toxic and showed a good cell affinity and morphology *in vitro* for 1 day.

## 1. Introduction

Human bone is composed mainly of collagen (20%), calcium phosphate (69%), and water (9%). The chemistry of the human bone is similar to hydroxyapatite (HA), which is composed of both calcium and phosphate at a ratio of 1 : 1.67 [1]. HA ceramic is one of the most widely used calcium phosphate materials in dental and medical fields. HA has the excellent biocompatibility with living bone tissue and does not cause defensive body reactions. Therefore, HA has attracted considerable attention as an alternative material in bone for several decades. On the other hand, the poor mechanical properties of HA precludes its use as a replacement with load-bearing applications [2].

The fracture toughness of the cortical bone of humans is 2–12 MPa·m^1/2^ whereas that of HA is only 1 MPa·m^1/2^. Therefore, it is essential to improve the mechanical properties, particularly the fracture toughness of HA. Some granular and fibrous additives made from polymers, glasses, and ceramics have been added to HA to improve their mechanical properties [3–5]. Recently, the use of the carbon nanotubes (CNTs) as reinforcement in the ceramic matrix to enhance the mechanical properties was suggested [6].

CNT is divided into single-walled CNTs (SWNTs) and multiwalled CNTs (MWNTs). SWNTs are a single rolled up graphene sheet but MWNTs are composed of concentric cylinders by graphene sheets of several layers. Both CNTs have many good properties, such as low density (2.1 g/cm^3^), high aspect ratio (1,000–10,000), and remarkable mechanical, thermal, and electronic properties for reinforcement [6, 7].

In principle, SWNTs are more effective as a reinforcement than MWNTs because the inner layers of the MWNT contribute a little in carrying a load, so the stiffness for a given volume fraction of tubes is reduced [8]. However, MWNTs are used as reinforcement in practice because SWNTs are considerably more expensive than MWNTs and there is little difference in other characteristics. Many studies have been reported for the use of MWNT as an additive in various types of ceramics. Nanosize HA composites with MWNT were studied with an aqueous precipitation process to examine the possible application as a scaffold for bone regeneration [9]. Agarwal group evaluated the effect of CNT addition to plasma-sprayed HA coating on its tribological behavior and biocompatibility of the coating and the cytotoxicity of CNT-containing HA wear debris [10].

The dispersion of CNTs is the largest problem impeding the use of CNTs as reinforcements in a ceramic matrix. Although CNTs have a dispersion problem, they are still good materials for reinforcement. Because CNTs have a tendency of making bundles, a range of methods, such as nanoscale dispersion (NSD), surface treatment, and heterocoagulation, have been developed for solving the dispersion problem [11–13]. If CNTs exist as agglomerate forms in a ceramic matrix, they would not toughen the matrix effectively.

In normal sintering methods, a high sintering temperature and long sintering time are needed for the fully dense consolidation of HA. On the other hand, such sintering condition results in extreme grain growth and the thermal decomposition of HA. These results cause a decrease in the mechanical properties [14] and alter the biodegradation rate of HA, respectively. To prevent these adverse results, spark plasma sintering (SPS) has been used to sinter CNT-HA composites. SPS allows the formation of a fine and uniform microstructure in a short period of time through the rapid heating rate by pulsed electrical discharge and high pressure application [15].

For biomaterial applications, the biological behavior of the CNT-reinforced HA composites is important. Several experiments with carbon-based biomaterials have already been done, and the biocompatibility of carbon materials has been proven [16, 17]. In addition, CNTs have recently been proven to be safe and effective as biomaterials by cell experiments [18].

In this study, experiments were carried out using MWNTs as reinforcements in HA composites because MWNTs and SWNTs have similar characteristics. In addition, the consolidation of MWNT-reinforced HA was conducted by SPS. The relative density, grain size, hardness, fracture toughness, and cell behavior of the SPS consolidated HA composites were evaluated in terms of the MWNT content and sintering temperature.

## 2. Experimental Procedure

### 2.1. Material Preparation

**Figure 1:**
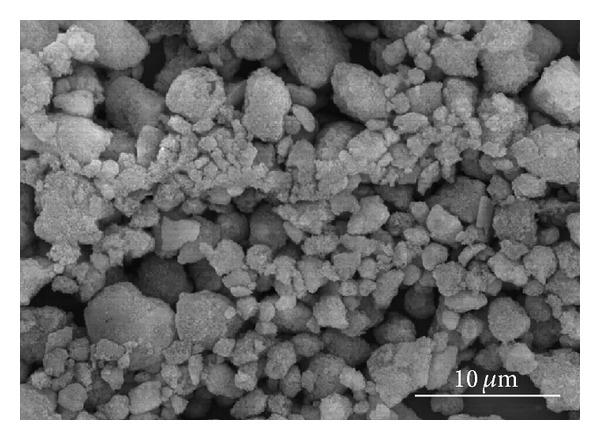
SEM of pure HA raw powder.

HA powder (Junsei Chemical, Japan) with a purity of 98% and a particle size of (3~9) *μ*m, as shown in Figure 1, was used in this experiment as a matrix. The dispersed MWNTs as reinforcements were purchased from Applied Carbon Nanotechnology (ACN), Korea. To obtain the homogeneous MWNTs and HA mixtures, the dispersion of the MWNTs was first dispersed mechanically by high energy ball milling with ZrO_2_ balls in an alcohol solvent for one hour. The dispersed MWNTs and HA powder were mixed using an ultrasonically mixing method. At that time, the HA powder was added gradually to the ultrasonically dispersing MWNT suspension in an alcohol solvent. The mixed content of the MWNTs in the HA matrix varied (0, 1, 3, 5, and 10 vol%). The mixture ink of the MWNTs and HA, which was dispersed ultrasonically in an alcohol solvent, was dried in a drying oven for 1 hour.

### 2.2. Spark Plasma Sintering

Consolidation of a mixture of MWNTs and HA was conducted using an SPS system (SPS. Dr. Sinter 5000, Fuji Electronic Industrial Co., Ltd, Japan). The mixed powder was poured into a graphite mold, 30 mm in diameter. A graphite sheet was placed between the mold and powder to prevent friction and easily separate the sintered sample from the graphite mold. The SPS sintering experiments of the samples were conducted uniaxially at 900, 1000, and 1100°C under vacuum conditions. The applied pressure was 20 KN up to 700°C and 45 KN at above 700°C. The sintering temperature was increased to 700°C at a heating rate of 100°C/min and then increased to between 900°C and 1100°C at a rate of 50°C/min. The holding time at each sintering temperature was 10 min. After holding, the furnace was cooled slowly at a rate of 5°C/min to prevent thermal shock of the sintered samples.

### 2.3. Characterization

The sintered disc type specimens were cut at 4 mm intervals and polished using SIC paper and a diamond suspension. The densities of the sintered samples were measured using the Archimedes method. The microstructures and grain sizes of the polished and fractured surfaces were characterized by scanning electron microscopy (SEM, Hitachi S-4800 using 15 kV and 10 *μ*A). The samples were polished with SiC sand paper, and then with a diamond suspension. The samples were then etched with a 0.05 N HCl solution and coated with osmium. The Vickers hardness and fracture toughness of the sintered test samples prepared by a high quality and smoothly polished specimen surface with no precracking were measured using the Vickers hardness test. The indentation on the polished specimens was carried out using a Vickers pyramidal microhardness indenter (MVK-H1, Akashi, Japan) at a load of 300 g for hardness and a 1 kg force for fracture toughness for 10 s. X-ray diffraction (XRD, D/max-2200, Rigaku, Japan) of each sintered body was carried out at 40 kV and 30 mA with a scan rate of 1°. The XRD patterns were used to verify the HA decomposition due to the SPS sintering conditions, and the MWNT content during the sintering process.

Osteoblast (MC3T3-E1) cell line was obtained from Korea Cell Bank. MEM alpha, fetal bovine serum (FBS), and penicillin G-streptomycin were acquired from Gibco, Japan. To assess the cytotoxicity of MWNT/HA composites (rod sample), the MWNT/HA composite bars were cut and fitted to a 6-well culture dish and subsequently immersed in a MEM alpha medium containing 10% fetal bovine serum (FBS), and 1% penicillin G-streptomycin. One mL of the MC3T3 cell solution (5 × 10^4^ cells/cm^2^) was added to the sample and incubated in a humidified atmosphere of 5% CO_2_ at 37°C for 1 day. After incubation, the supernatant was removed, washed twice with PBS, and fixed in a 2.5% glutardialdehyde aqueous solution for 20 min. The samples were then dehydrated in a critical point drier and finally sputter-coated with gold. The surface morphology of the samples was then observed by SEM [19].

## 3. Results and Discussion

**Figure 2:**
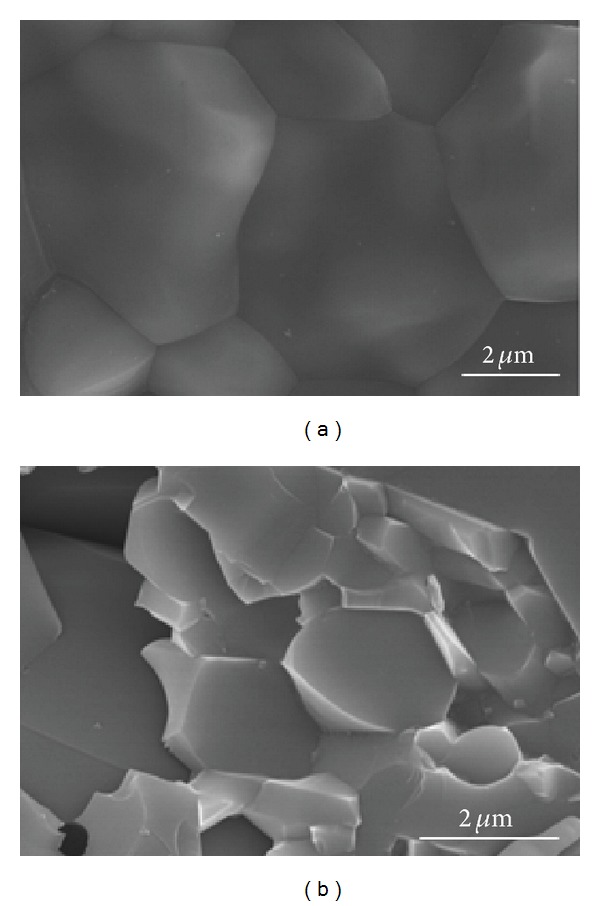
SEM of pure HA sintered at 1250°C for 2 hrs in air using a conventional electric furnace (a), and at 1100°C for 10 min using a spark plasma sintering system (b).

The sintering of pure HA was conducted at 1250°C for 2 hrs using an electric furnace in air (Figure 2(a)) and at 1100° under a pressure of 60 MPa for 10 min by SPS in a vacuum (Figure 2(b)). The measured relative densities of the pure HA sintered by conventional and SPS methods were approximately 99% and 98%, respectively.

The pure HA sintered by SPS showed less grain growth than the HA sintered by conventional sintering due to relatively lower sintering temperatures and shorter sintering time with a high pressure during sintering. SPS did not allow time for grain growth due to the rapid heating rate and short sintering time. The pressure of SPS also helped restrain grain growth. In this study, the mean grain size of the conventionally sintered pure HA at 1250°C for 2 hrs in air was approximately 6 *μ*m (Figure 2(a)), and that of SPS at 1100° for 10 min was approximately 1.6 *μ*m (Figure 2(b)).

**Figure 3:**
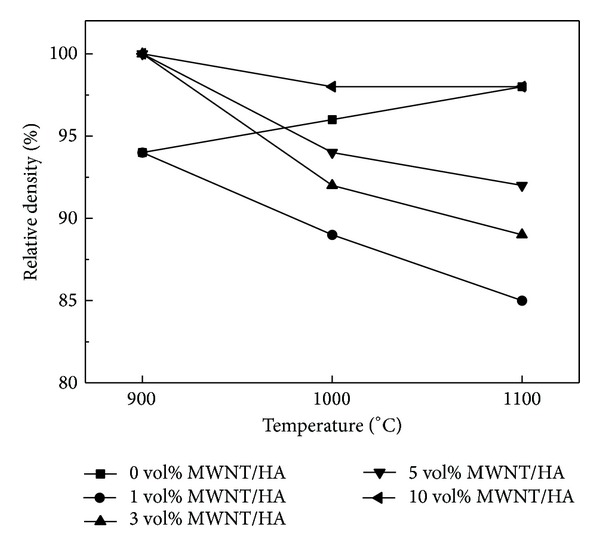
Effect of the MWNT content (volume %) on the relative density of sintered MWNT-reinforced HA composites.

**Figure 4:**
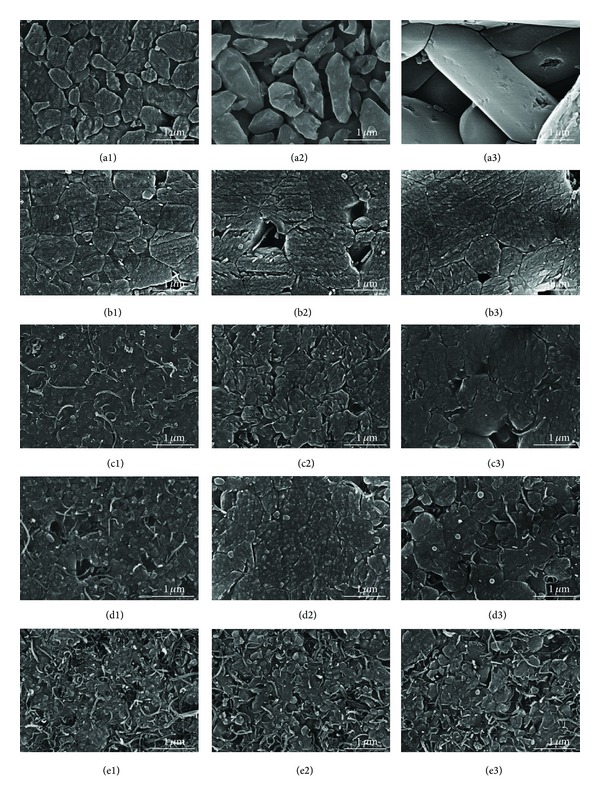
SEM images of the polished surfaces of sintered MWNT-reinforced HA composites; ((a1), (a2), (a3)) for pure HA; ((b1), (b2), (b3)) for 1 vol%; ((c1), (c2), (c3)) for 3 vol%; ((d1), (d2), (d3)) for 5 vol%; and ((e1), (e2), (e3)) for 10 vol% MWNT-reinforced HA composites. The numbers (1, 2, 3) represent the sintering temperatures (900, 1000, and 1100°C, resp.).

Figure 3 shows the relative densities of the MWNT/HA composites sintered by SPS. The relative density of the sintered pure HA increased with increasing sintering temperature from 94% at 900°C to 98% at 1100°C. On the other hand, the relative density of the MWNT-reinforced HA (MWNT/HA) composites sintered by SPS reached almost full density at 900°C and then decreased with increasing sintering temperature. The large difference in the thermal expansion coefficient between the MWNTs (0.99 × 10^−5^/°C) and HA (1.479 × 10^−5^/°C) resulted in a larger shrinkage difference between the MWNTs and HA in the sintered MWNT/HA composites during cooling. The larger shrinkage of the MWNTs in the MWNT/HA composites generated pores around the MWNTs. The difference in shrinkage increased with increasing sintering temperature. Therefore, more pores were observed at a higher sintering temperature (Figure 4).

The relative density of the MWNT/HA composites increased with increasing MWNT content. The higher thermal conductivity of the MWNTs (2890 W/mK) than that of HA (1.25 W/mK) resulted in a faster and more homogeneous temperature distribution in the sample during sintering and a higher sintering density at low sintering temperatures. With increasing MWNT content, the homogeneous temperature distribution was higher and densification occurred at a lower sintering temperature. Deng et al. reported that the relative density of the CNT/aluminum composites was increased by the addition of CNTs at the same temperature. They suggested that CNT addition could fill up the microvoids in the aluminum matrix resulting in an increase in the relative density of the composites [20].

**Figure 5:**
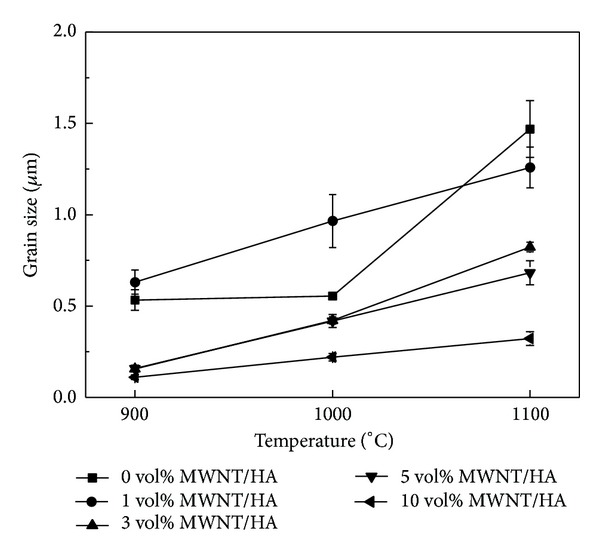
Effect of the MWNT volume % on the grain size of the sintered MWNT-reinforced HA composites.

Grain growth was analyzed from the polished surfaces of the SPS consolidated MWNT/HA composites by SEM, as shown in Figure 4.  Figure 5 shows the grain size of the sintered MWNT/HA composites. In Figure 4, the small letters (a to f) represent the MWNT content, that is, 0, 1, 3, 5, and 10 vol%, and the numbers (1, 2, 3) stand for the sintering temperatures, such as 900, 1000, and 1100°C.

The grain size of the MWNT/HA composite sintered by SPS was smaller than that of pure HA sintered by SPS. The mean grain size of pure HA was 0.5 *μ*m at 900°C and 1.6 *μ*m at 1100°C, but that of the MWNT/HA composites was 0.1~0.6 *μ*m at 900°C and 0.3~1.2 *μ*m at 1100°C, depending on the MWNT content. The mean grain size of the MWNT/HA composites decreased with increasing MWNT content.

The mean grain size of the MWNT/HA composites increased gradually with increasing sintering temperature. The MWNT/HA obtained sufficient energy for grain growth at a relatively low temperature because of the higher thermal and electrical conductivity (2890 W/mK and 2 × 10^4^ S cm^−1^) of the MWNTs, compared to that (1.25 W/mK and 7 × 10^−7^ S cm^−1^) of HA [21]. In addition, the MWNTs in the HA composites restrain grain growth during the sintering process and expedite grain refinement because the MWNTs are located in the grain boundaries of the HA matrix [22]. Therefore, the grain size of the MWNT/HA composites decreased and became more uniform with increasing MWNT content.

On the other hand, undispersed or agglomerated MWNTs in the MWNT/HA composites were found. The existence of agglomerated MWNTs resulted in the formation of large pores. The formation of large pores increased with increasing sintering temperature and MWNT content, as shown in Figure 4(e). However, there are no large pores in the vicinity of the region of well-dispersed MWNTs in Figure 4(e). This typical result showed why the dispersion process of the MWNTs, SWNT, and graphene is important for their use as reinforcement in the matrix regardless of the polymer or ceramic.

**Figure 6:**
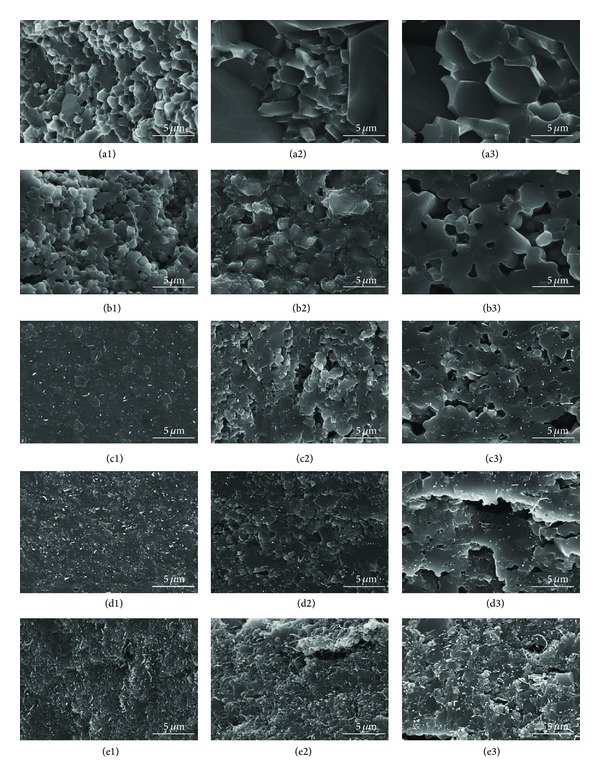
SEM images of the fractured surfaces of MWNT-reinforced HA composites. ((a1), (a2), (a3)) pure HA; ((b1), (b2), (b3)) 1 vol%; ((c1), (c2), (c3)) 3 vol%; ((d1), (d2), (d3)) 5 vol%; and ((e1), (e2), (e3)) 10 vol% MWNT-reinforced HA composites. The numbers (1, 2, 3) represent the sintering temperatures (900, 1000, and 1100°C, resp.).

**Figure 7:**
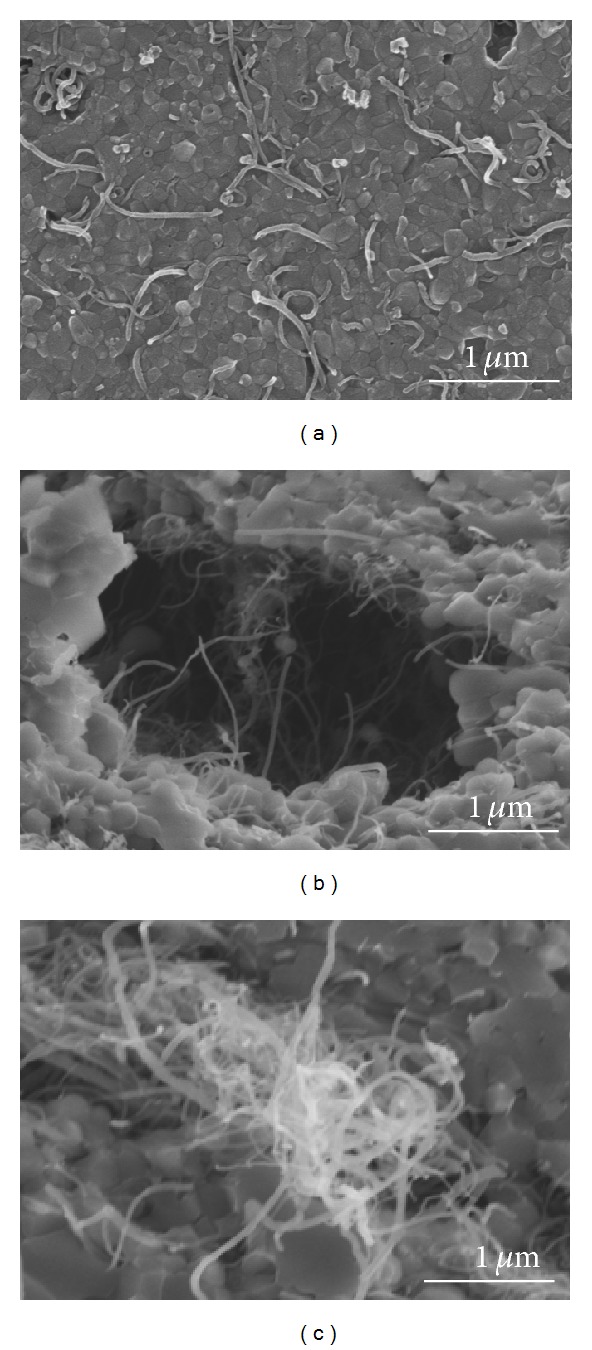
SEM images of the polished surface (a) and fractured surfaces (b) (c) of 10 vol% MWNT/HA composites sintered at 900°C.

Figure 6 shows the fractured surface of the MWNT/HA composites consolidated by SPS. As shown in Figure 6, this result clearly shows the effects of the MWNTs in the MWNT/HA composites on grain growth with increasing MWNT content. Figure 6 shows the pull-out toughening of the MWNTs in the fractured surface of the MWNT/HA composites. Therefore, an improvement in mechanical properties can be predicted by the toughening mechanism. No large or small pores were found in the well-dispersed MWNT region (Figure 7(a)). On the other hand, agglomerates of undispersed MWNTs were found and were always accompanied by large pores caused by the agglomeration of MWNTs (Figures 7(b) and 7(c)). The toughening of the MWNT/HA composites suggests that MWNTs are suitable as reinforcements in the HA matrix although the formation of the large pores might underline the mechanical properties [23].

**Figure 8:**
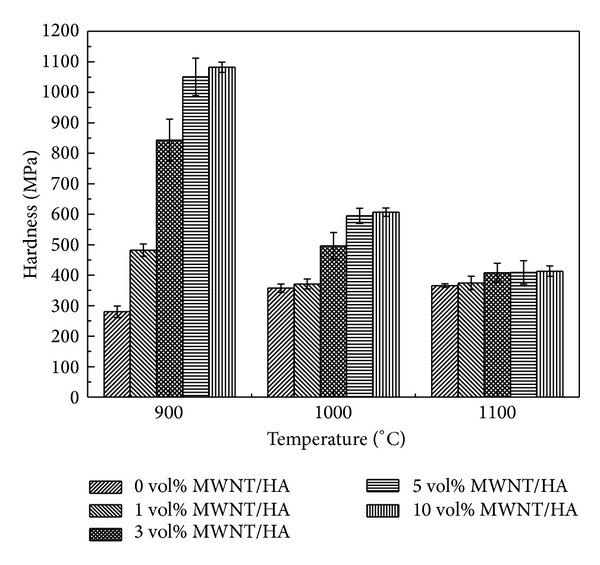
Hardness versus sintering temperature of the sintered MWNT-reinforced HA composites.

**Figure 9:**
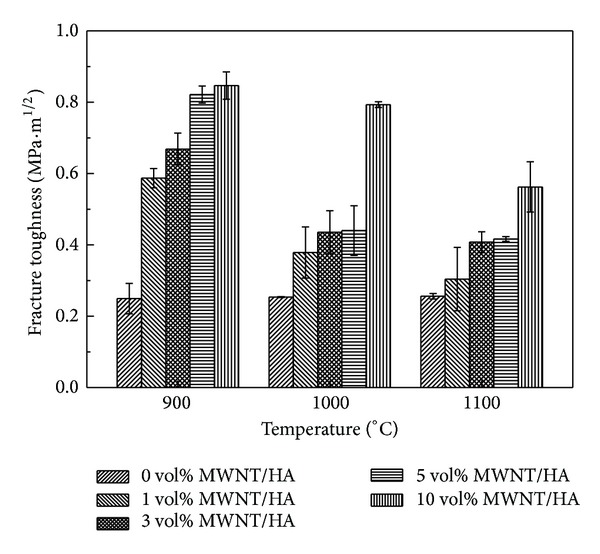
Fracture toughness versus sintering temperature of the sintered MWNT-reinforced HA composites.

Figures 8 and 9 show the Vickers hardness and fracture toughness of the MWNT/HA composites as a function of the sintering temperature, respectively. The mechanical properties of the sintered materials are related to the relative density. In this study, both the hardness and fracture toughness of the MWNT/HA composites were related to their relative densities and increased with increasing density (as shown in Figure 3). In all the MWNT/HA composites, the hardness and fracture toughness reached a maximum at 900°C and then decreased with further increases in sintering temperature. On the other hand, the hardness and fracture toughness of the pure HA increased slightly with increasing sintering temperature because the relative density increased with increasing sintering temperature.

The hardness and fracture toughness of the MWNT-reinforced HA increased with increasing MWNT content (Figures 8 and 9). The smaller grain size and slightly higher density are believed to result in higher hardness and fracture toughness. The hardness and fracture toughness of the 10 vol% MWNT-reinforced HA sintered at 900°C were 1.1 GPa and 0.85 MPa·m^1/2^, respectively. On the other hand, that of the pure HA sintered at 900°C were 280 MPa and 0.25 MPa·m^1/2^, respectively. This shows that despite the presence of large pores caused by undispersed MWNTs, the addition of MWNTs to HA increased the hardness and fracture toughness by approximately 3~4 times. This suggests that the MWNTs are good candidates as reinforcement for strengthening the ceramic matrix. The Vickers hardness and fracture toughness were calculated using the following equations:

(1) $$\begin{array}{c} \text{HV}=1.8544\frac{P}{d^{2}}, \end{array}$$

where *P* is the applied test load in N, *d* is the arithmetic average of the two diagonal lengths of the resultant impression in mm, and 1.8544 is the geometrical constant of the diamond pyramid [24]:

(2) $$\begin{array}{c} K_{\text{IC}}=0.0726\frac{P}{c^{1.5}}, \end{array}$$

where *P* is the applied load (N) and *c* is the crack length from the center of the indent to the crack tip (m) [25].

HA was reinforced by toughening mechanisms, such as pull-out by the MWNTs and the small grain size. The mechanical strength of the MWNT/HA composites was also related to the grain size. The grain size refinement by MWNT addition is the main reason for the improvement in the mechanical strength of HA.

**Figure 10:**
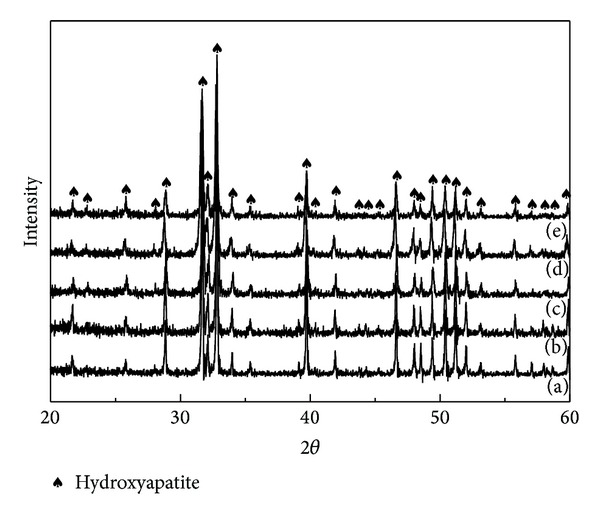
XRD patterns of the MWNT-reinforced HA composites sintered at 900°C: (a) 0 vol.% (pure HA); (b) 1 vol.%; (c) 3 vol.%; (d) 5 vol.%; (e) 10 vol.% MWNT.

SPS is used to consolidate the MWNT/HA composites and avoid the decomposition of HA to HA and TCP during conventional sintering [26]. The thermal decomposition of HA decreases the mechanical properties and increases the degradation rate. XRD was conducted to assess the possibility of phase decomposition.  Figure 10 shows the XRD pattern of the MWNT-reinforced HA composites sintered by SPS at 900°C. All the XRD patterns of the MWNT/HA composites consisted of HA peaks with no MWNT peaks. The lack of peaks of MWNT was attributed to the relatively strong intensity of the HA peaks. Owing to SPS with a lower sintering temperature and shorter sintering time, no decomposition of MWNT/HA composites occurred, as confirmed by XRD.

**Figure 11:**
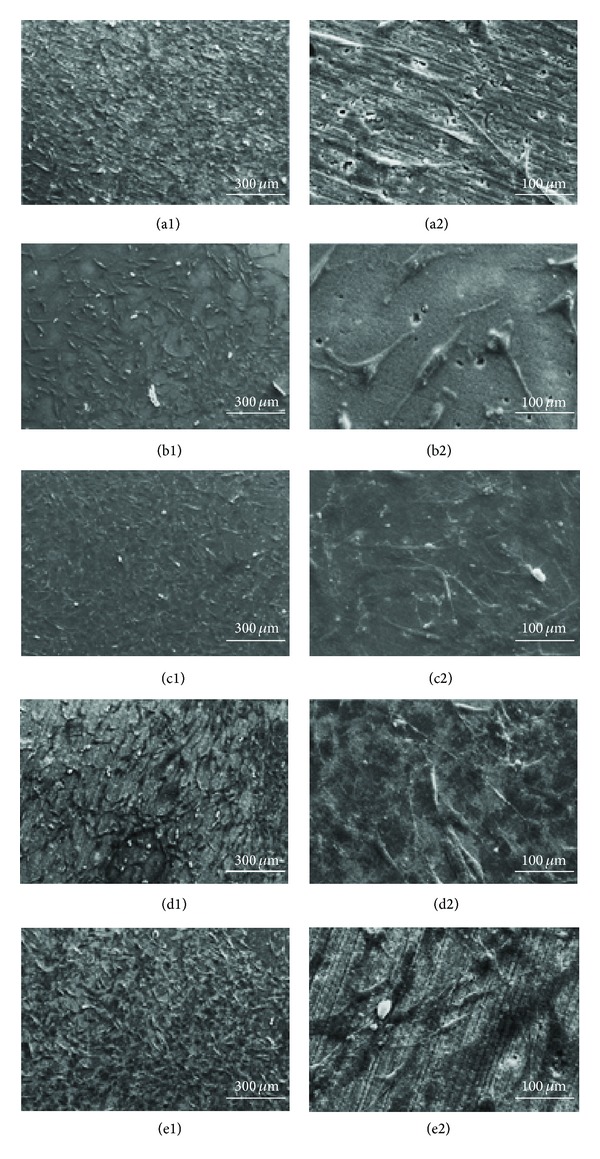
SEM images of osteoblastic cells on the sintered MWNT-reinforced HA composite after culturing for 1 day: (a1) pure HA; (b1) 1 vol%; (c1) 3 vol%; (d1) 5 vol%; and (e1) 10 vol% MWNT-reinforced HA composites, and (a2), (b2), (c2), (d2), and (e2) represent the high magnification images of (a1), (b1), (c1), (d1), and (e1), respectively.

To examine the possibility of biomaterials applications, the cell behavior of the MWNT-reinforced HA composite and pure HA was compared.  Figure 11 shows the results of the *in vitro *study of the osteoblastic cells on the MWNT-reinforced HA after culturing for 1 day. The osteoblastic cells were attached well to the surface of all composites. The MWNTs did not show the adverse effect on the cell attachment, even at the high content of MWNT (10 vol%). In general, the MWNT-reinforced HA composites were nontoxic and showed a good cell affinity and morphology.

## 4. Conclusion

Pure HA and 1, 3, 5, and 10 vol% MWNT-reinforced HA were consolidated by SPS. The relative density of HA increased with increasing sintering temperature, but that of the MWNT/HA composite reached almost full density at 900°C, and then decreased with increasing sintering temperature. Owing to the excellent thermal conductivity of the MWNTs, the relative density of the MWNT/HA composites increased with increasing MWNT content.

The grain size of the MWNT/HA composites decreased with increasing MWNT content and increased with increasing sintering temperature. The MWNTs located in the grain boundaries in the HA composites retard grain growth during the sintering process and expedite grain refinement. The pull-out toughening of the MWNTs of the MWNT/HA composites was observed in the fractured surface. Therefore, the improvement of the mechanical properties can be predicted. On the other hand, the existence of undispersed or agglomerate MWNTs in the MWNT/HA composites resulted in the formation of large pores. The formation of large pores increased with increasing sintering temperature and MWNT content.

The Vickers hardness and fracture toughness of the MWNT/HA composites reached a maximum at 900°C and then decreased with increasing sintering temperature. The hardness and fracture toughness increased with increasing MWNT content. This shows that despite the presence of large pores caused by undispersed MWNTs the addition of MWNT to HA increased the hardness and fracture toughness by approximately 3~4 times. This can explain why MWNTs are good candidates as reinforcement for strengthening the ceramic matrix.

XRD of MWNT/HA composites sintered by SPS at 900°C revealed HA peaks without decomposed TCP peaks. The *in vitro *study for 1 day showed that the osteoblastic cells were attached well to the surface of all the MWNT/composites. This confirmed that the MWNTs in the MWNT-reinforced HA composites were nontoxic and did not result in the adverse cell affinity and morphology.

MWNTs are good candidates as reinforcement for strengthening HA matrix composites, and the MWNT/HA composite can be used for load bearing or nonload bearing biomaterial applications.
